# Therapeutic potential of ayahuasca in grief: a prospective, observational study

**DOI:** 10.1007/s00213-019-05446-2

**Published:** 2020-01-14

**Authors:** Débora González, Jordi Cantillo, Irene Pérez, Magí Farré, Amanda Feilding, Jordi E. Obiols, José Carlos Bouso

**Affiliations:** 1 International Center for Ethnobotanical Education, Research and Services (ICEERS), Carrer de Sepúlveda, 65, Office 2, 08015 Barcelona, Spain; 2 PHI Asociation, Passeig del Calvell 35-37, 08005 Barcelona, Spain; 3 Clinical Pharmacology Unit, Hospital Universitari Germans Trias i Pujol and Institut de Recerca Germans Trias i Pujol (IGTP), Ctra. de Can Ruti s/n, 08916 Badalona, Spain; 4 Department of Pharmacology, Therapeutics and Toxicoloy, Universitat, Autònoma de Barcelona, Avinguda de Can Doménech, 08193 Cerdanyola del Vallés, Spain; 5 The Beckley Foundation, Beckley Park, Oxford, OX3 9SY UK; 6 Department of Clinical and Health Psychology, Universitat Autònoma de Barcelona, Bellaterra Campus, Building B, Office B5/016b, 08193 Cerdanyola del Vallés, Spain

**Keywords:** Ayahuasca, Shipibo, Death, Bereavement, Grief, Psychopathology, Quality of life, Experiential avoidance, Acceptance, Decentering

## Abstract

**Rationale:**

Recent studies have assessed the therapeutic potential of ayahuasca for the treatment of depression with promising preliminary results**.**

**Objectives:**

Here, we examine the course of grief over 1 year of follow-up in a bereaved sample that attended a center in Peru to participate in indigenous Shipibo ayahuasca ceremonies. We also explore the roles of experiential avoidance and decentering as mechanisms of change.

**Methods:**

Bereaved participants who attended the ayahuasca center responded to an online survey that included the Texas Revised Inventory of Grief, Symptom Assessment-45, WHO Quality of Life-Bref, Acceptance and Action Questionnaire, and Decentering. Baseline assessment was completed by 50 individuals (T0). Of these, 39 completed the post-assessment at 15 days (T1), 31 at 3 months (T2), 29 at 6 months (T3), and 27 at 12 months (T4) after leaving the retreat. Pearson’s analysis was performed to examine the relationship between the severity of grief and mechanisms of change during the period of T0 and T1.

**Results:**

A significant decrease in Texas Revised Inventory was observed at all time points (T1: Cohen’s *d* = 0.84; T2: Cohen’s *d* = 1.38; T3: Cohen’s *d* = 1.16; T4: Cohen’s *d* = 1.39). We found a relationship between experiential avoidance (*r* = 0.55; *p* < .01), decentering (*r* = − 0.47; *p* < .01), and a reduction in the severity of grief.

**Conclusions:**

Our results suggest that the ceremonial use of ayahuasca has therapeutic value by reducing the severity of grief. Acceptance and decentering are both psychological processes that mediate the improvement of grief symptoms.

**Electronic supplementary material:**

The online version of this article (10.1007/s00213-019-05446-2) contains supplementary material, which is available to authorized users.

## Introduction

The word “ayahuasca” is derived from the Quechua language, *aya* means “dead person, spirit, soul, or ancestor” and *huasca* means “rope or vine” (Metzner, 2005). Ayahuasca is a natural plant concoction used by indigenous groups throughout the Amazon Basin in traditional medicine and spiritual rituals (Schultes and Hofmann, 1992). The concoction is usually prepared by boiling the stems of the *Banisteriopsis caapi* vine with the leaves of the *Psychotria viridis* shrub, or *Diplopterys cabrerana* leaves (Schultes and Hofmann, 1992). *B. caapi* is rich in beta-carbolines, which have monoamine oxidase-inhibiting properties, the main pharmacological mechanism of some antidepressants currently used in clinical practice (Meister et al. 2016). *P. viridis* and *D. cabrerana* contain the tryptamine N,N-dimethyltryptamine (DMT) that acts as an agonist of 5-HT-2A and sigma-1 receptor sites, which is also associated with antidepressant, anxiolytic, and psychoactive effects (Fontanilla et al. 2009; Domínguez-Clavé et al. 2016).

The evidentiary literature on ayahuasca’s pharmacological safety among healthy volunteers (Dos Santos et al. 2013, 2012; Riba et al. 2003), and the lack of dose tolerance and addictive potential (Fábregas et al. 2010), has triggered extensive scientific research into this concoction during the last two decades. Single-photon emission computed tomography (SPECT) techniques have shown ayahuasca to produce an increased blood perfusion in the left nucleus accumbens, right insula and left subgenual area, brain regions implicated in the regulation of mood and emotions (Sanches et al. 2016). Functional magnetic resonance imaging have shown a significant decrease in the activity of the Default Mode Network (DMN) (Palanho-Fontes et al. 2015), a brain network that is hypperactivated in patients with major depressive disorder (MDD), which is associated with maladaptive rumination and self-referential processes (Hamilton et al. 2011; Sheline et al. 2009). Moreover, ayahuasca enhances creative divergent thinking, a psychological process underlying psychological flexibility that is relevant to the success of cognitive therapy (Kuypers et al. 2016). Regular ayahuasca ceremony participants have performed well on neuropsychological tests (Bouso et al. 2012, 2015) and show low levels of psychopathological symptoms (Bouso et al. 2012, 2015; Barbosa et al. 2009). Rapid anxiolytic and antidepressant effects have been demonstrated in animals (Da Silva et al. 2018; Cameron et al. 2018) and human beings (Dos Santos et al. 2007; Palhano-Fontes et al. 2019; Osorio et al. 2015). A recent study supporting these results observed a modulation of salivary cortisol in major depression treatment-resistant patients, where concentrations similar to those of healthy volunteers were reached 48 h after a single dose of ayahuasca (Galvão et al., 2018).

Studies about the mediating psychological processes underpinning ayahuasca’s therapeutic potential are also growing. A previous study found that 4 ayahuasca sessions could be as effective at reducing experiential avoidance (or, formulated positively, at promoting the acceptance of negative feelings and thoughts) as 8 weeks of mindfulness-based stress reduction (MBSR) (Soler et al. 2018). Decentering, defined as the ability to observe one’s thoughts and feelings in a detached manner (Fresco et al. 2007), also improves 24 h after drinking ayahuasca (Soler et al. 2016). This ability seems to increase even more in people who drink ayahuasca regularly (Franquesa et al. 2018), as long as they do not possess borderline personality traits (Domínguez-Clavé et al., 2019). Other studies have found improvements in other mechanisms of change that are also classic goals of mindfulness training. This is the case for positive self (Franquesa et al. 2018), inner reactivity, and the judgmental processing of experiences (Soler et al. 2016).

Until now, only one exploratory observational study has found preliminary evidence regarding the therapeutic potential of ayahuasca for grief related to the death of a loved one (González et al. 2017). Bereavement, defined as the period of grief and mourning after the death of a love one, is a universal condition and one of the most painful experiences that humans face. Feeling stunned or shocked, emotional numbness, mistrust of others, bitterness over the loss, confusion about one’s role in life, a diminished sense of self, difficulty accepting the loss, and moving on with life are normal reactions when bereaved in the aftermath of a death of a love one (Prigerson et al. 2009). Prolonged Grief Disorder (PGD) has recently been included in the latest version of the International Classification of Disease (ICD-11) (World Health Organization, 2018), with an estimated prevalence of 9.8% among bereaved individuals (Lundorff et al. 2017). Core PGD symptoms include pervasive yearning for the deceased and persistent preoccupation with the deceased, accompanied by intense emotional pain for, at minimum, 6 months post-loss. In addition, PGD is characterized by cognitive, emotional, and behavioral symptoms that disrupt the individual’s ability to function in different contexts (World Health Organization, 2018). Research has found that symptoms of PGD are associated with impairment of the bereaved person’s familiar, social and occupational functioning to a similar extent as found in depression and post-traumatic stress disorder (Prigerson et al. 2009; Jordan and Litz 2014; Maercker et al. 2013). Drugs alone have not been shown to effectively treat PGD symptoms (Zisook and Shear 2009; Shear et al. 2016). The latest meta-analysis of controlled trials found that the mean effect sizes of the current psychotherapeutic interventions are smaller than those of treatments for other psychiatric disorders (Johannsen et al. 2019; Wittouck et al. 2011). Moreover, the results from grief interventions on non-pathological grief provide inconsistent support for their effectiveness (Wittouck et al. 2011).

The present study investigated the long-term effects of ayahuasca in participants who are grieving the death of a loved one, following a sample of subjects with a history of grief who willingly attended ayahuasca ceremonies at an ayahuasca center in Peru. The primary outcome variable was the severity of grief. Secondary variables were psychopathological symptoms, quality of life, experiential avoidance, and decentering. We hypothezised that the severity of grief would be reduced after drinking ayahuasca at the center. Second, we hypothezised that psychopathological symptoms, quality of life, and both psychological processes would also improve after drinking ayahuasca. Given that previous studies have shown that acceptance and decentering are psychological processes underlying ayahuasca’s effects, and given that both constructions play an important protective role in psychopathology models, we finally hypothesized that the improvement of both psychological processes would mediate the reduction in the severity of grief.

## Methods

This study is part of a broader study that aims to prospectively assess the long-term effects of ayahuasca on well-being and quality of life, as well as psychopathological symptoms in different subsamples with depression, anxiety, post-traumatic stress disorder, and grief (data are currently pending publication). In the present article, only the data for the grief subsample has been included and analyzed.

### Participants and procedure

Participant enrollment was carried out between 2015 and 2017. During 2018, the follow-up period of 1 year was concluded. Participants were invited to participate in the study through the website of the Temple of the Way of Light (Temple of the Way of Light 2016), after having booked their stay at the center. This center offered retreats of 9, 12, or 30 days, where participants drank ayahuasca between 4 and 9 times in a ceremonial context under the guidance of Shipibo Curanderos (traditional healers). The retreat also offers optional yoga classes, floral and steam baths, and integration circles concerning ayahuasca experiences. Information about the retreats is available online (Temple of the Way of Light 2016). Despite the fact that these programs were not specifically developed to treat grief symptoms, our goal was to reach a sufficient number of participants who were suffering from the loss of a loved one when they attended the retreat.

A total of 50 participants were elegible for the analysis. The screening process involved two stages. First, to be accepted by the center, which requires individuals to be 18 years of age or older. People with previous clinical disorders (i.e., psychosis, depersonalization, or mania), people who were taking certain medications (i.e., MAOIs or SSRIs), people with a heart condition, those with chronic high blood pressure, and pregnant women were not accepted (Temple of the Way of Light 2019). Second, to respond to a short online questionnaire including the following questions: (a) Please indicate the exact date when you will attend to the retreat; (b) please mark “yes,” “uncertain,” or “no” to the following questions: are you currently going through a period of (b.1) depression; (b.2) anxiety; (b.3) post-traumatic stress; (b.4) grief related to the death of a loved one; (c) can you understand with ease everything you read in the English language? Participants were included in the study if they affirmed feeling grief symptoms related to the death of a loved one (regardless of whether they claim to be going through a period of depression, anxiety, or trauma) and they affirmed having an adequate knowledge of the English language.

Once participants were included in the study, assessments were done 15 days before attending the retreat (T0) and 15 days (T1), 3 months (T2), 6 months (T3), and 12 months (T4) after leaving the retreat. Participants who were included in the study more than 2 months before attending the center were invited to respond to an additional assessment 2 months before entering the center (T-1) in order to control for the effect of time on outcomes in a subgroup.

All questionnaires were administered online using LimeSurvey (version 1.92.), which allowed for the collection and preservation of the data on a secure server that is accessible only to the researchers via a password.

### Measures

#### Texas Revised Inventory of Grief

This questionnaire is a self-report measure designed to quantify the severity of grief (Faschingbauer 1981). In this study, only the Present Feelings Scale was used to measure a person’s emotional state at the time of the questionnaire’s completion. The questionnaire comprises a total of 13 items scored on a 5-point Likert-type scale (1 = completely false to 5 = completely true). The Texas Revised Inventory of Grief (TRIG) is score by calculating the average scores for the subscale. Higher scores indicate a greater severity of grief. The scale has been shown to have adequate psychometric properties with Cronbach’s coefficient alpha value of .90 in the present feelings scale.

#### Symptom Assessment-45 Questionnaire

This questionnaire is a self-report clinical measure designed to assess psychiatric symptoms in 9 different symptom domains, as well as to provide a general measure of overall psychiatric distress (GSI) (Davison et al., 1997). It contains 45 items, which fall into the following scales: Anxiety, Hostility, Obsessive-Compulsive, Phobic Anxiety, Somatization, Depression, Interpersonal Sensitivity, Paranoid Ideation and Psychoticism. Examinees are asked to indicate how much the particular problem has bothered them during the past 7 days on a 5-point Likert-type scale (0 = not at all to 4 = extremely). The Symptom Assessment-45 Questionnaire (SA-45) has been normed using both non-patient (*N* = 1621) and clinical samples (*N* = 13.844) (Maruish et al. 1998). Each of the samples included norms for both adolescent and adult males and females. Raw scores are transformed into standard *T* scores (*M* = 50, SD = 10). The scale has been shown to have adequate psychometric properties with Cronbach’s coefficient alpha values ranging from 0.69 to 0.92.

#### World Health Organization Quality of Life-BREF

This questionnaire is a shorter version of the original WHOQOL-100 (Whoqol Group 1998). This questionnaire was designed to assess quality of life in four domains: Physical Health, Psychological Health, Social Relationships, and Environment. For the purpose of this study, only the first three scales were used. The questionnaire comprises a total of 24 items, scored on a 4–20 scale, with higher scores indicating a better quality of life. The scale has been shown to have adequate psychometric properties with Cronbach’s coefficient alpha values ranging from 0.68 to 0.82 (Skevington et al. 2004).

#### Acceptance and Action Questionnaire

This is a unidimensional scale that assesses the construct referred to variously as experiential avoidance or, when it is positively formulated, acceptance (Bond et al. 2011). Experiential avoidance refers to avoidance of the experience of internal events, such as attempts to suppress or control emotions (Hayes 1987). Participants indicate their response to each question using a 7-point Likert-type scale (1 = never true to 7 = always true). The Acceptance and Action Questionnaire (AAQ-II) is score by adding together all the items, with higher scores corresponding to greater experiential avoidance. This questionnaire has been shown to have adequate psychometric properties with a Cronbach’s coefficient alpha value of 0.82.

#### EQ-Decenetering

This scale assesses decentering, defined as the ability to observe one’s thoughts and feelings in a detached manner (Fresco et al. 2007). The original scale was called the Experiences Questionnaire (EQ), in which participants rate items on a 7-point Likert-type scale (1 = never to 7 = all the time), assessing decentering and rumination (Fresco et al. 2007). Based on the psychometric characteristics of the original scale, which showed poor loadings for other items placed on the rumination factor and a robust structure for the decentering factor, only the EQ-Decentering scale was used for the present study. This is an 11-item self-report measure of decentering. Items are rated on a 5-point Likert scale (1 = never to 5 = always). The original EQ showed a high reliability coefficient with Cronbach’s alpha value of 0.90.

#### Bereavement adjustment questionnaire

An additional questionnaire was designed to explore bereavement adjustment during follow-up assessments (Appendix 1). These questions covered the following: participants’ subjective perceptions about the evolution of their clinical status; the persistent long-term harms and benefits derived from ayahuasca intake during their stay at the center; and participants’ use of ayahuasca, medication and psychotherapy to treat grief symptoms after leaving the center (at T1, T2, T3, and T4).

### Ayahuasca samples analyses

We obtained samples of two different ayahuasca batches prepared by Shipibo shamans at the Temple of the Way of Light along the period of the study. The ayahuasca was prepared by boiling the stems of *B. caapi* (rich in harmine, tetrahydroharmine, and harmaline) combined with the leaves of *P. viridis* (rich in N,N-dimethyl-tryptamine) for several hours. Analyses were carried out by Energy Control (energycontrol-international.org) using liquid chromatography-mass spectrometry (LC-MS). One ayahuasca sample contained 2 mg/ml N, N-DMT, 2 mg/ml of harmine, 0.37 mg/ml of harmaline, and 1 mg/ml of tetrahydroharmine. The other sample contained 2 mg/ml N, N-DMT, 2 mg/ml of harmine, 0.65 mg/ml of harmaline, and 2 mg/ml of tetrahydroharmine. No other psychoactive compounds were detected.

### Statistical analysis

Baseline sample characteristics, setting variables, and bereavement adjustment over time were analyzed descriptively. Changes in primary and secondary outcome variables between each time point and baseline were tested using Student’s paired two-sided *t* test or the Wilcoxon paired test, as appropriate. Bonferroni corrections were applied to correct for multiple comparisons. A 95% CI for the mean of the primary outcome at all time point assessments was included to further clarify plausible values. Effect sizes were calculated using Cohen’s *d*. Effect sizes were defined as large up to 0.8 (*d* > 0.8) (Cohen 1988). To examine the relationship between experiential avoidance (AAQ-II), decentering (EQ-D), and the severity of grief (TRIG), we used Pearson’s linear correlation coefficient. Correlation coefficients between 0.30 and 0.70 were considered moderate.

Additional subgroup analysis was performed separately for patients who responded to T-1, T0, and T1 assessments. We used the chi-square test for categorical variables and Fisher-Snedecor for continuous variables to evaluate homogeneity between the group who completed the follow-up at T1, and a subgroup that completed an additional assessment at T-1. Subgroup analyses were conducted using repeated measures analysis of variance (ANOVAs) with post hoc Bonferroni-correction, in order to examine differences in primary and secondary outcomes between the periods of T-1 to T0 and T1 to T0.

In this study, *p* values < 0.05 were considered statistically significant. Statistical analysis was performed using Statistical Package for the Social Sciences (SPSS for Windows, version 20).

## Results

### Participants

**Fig. 1:**
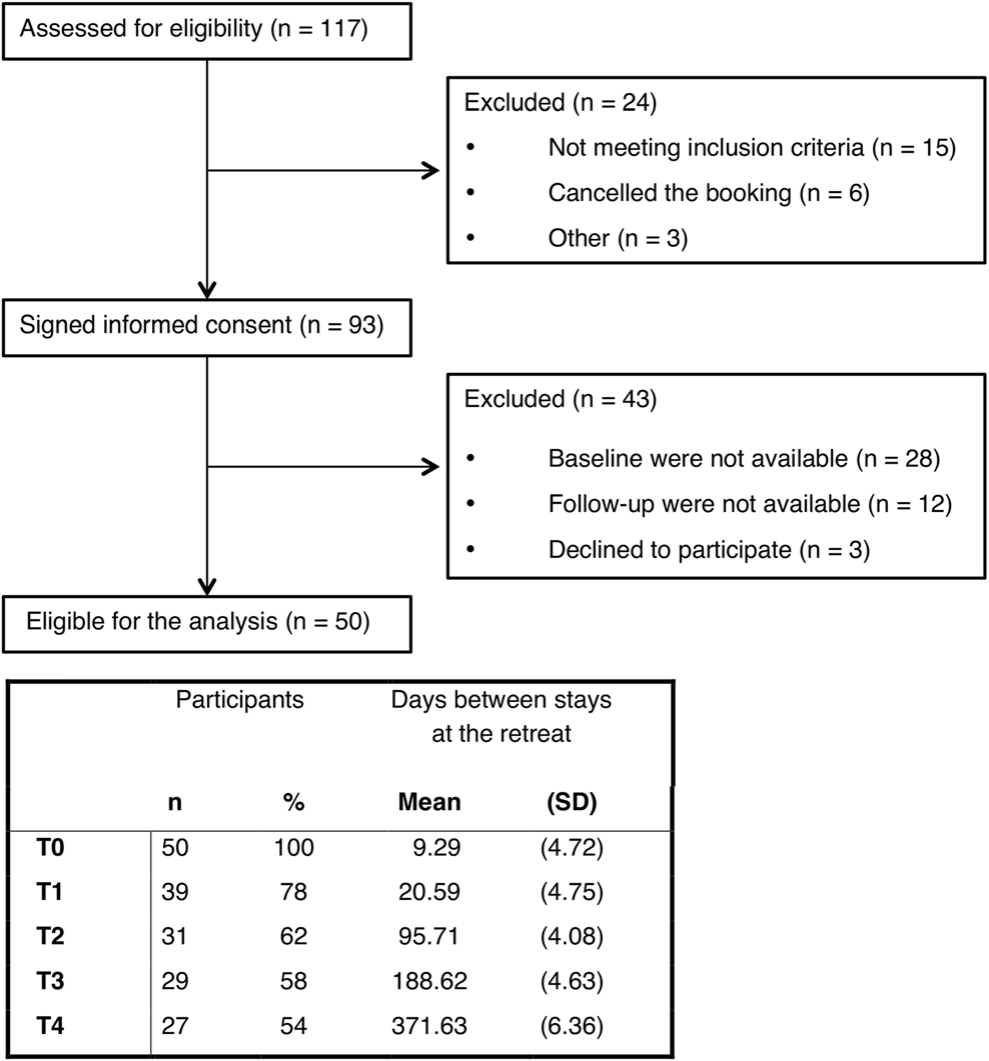
Participant flow throughout the study

Out of 117 participants who were assessed for eligibility, 79.49% participants signed the informed consent form. However, 30.1% participants were excluded because baseline data were not available, 12.91% were excluded because no follow-ups were available, and 3.23% expressed a desire to abandon the study. Finally, 50 participants were elegible for the analysis, having completed the baseline assessment and, at least, one follow-up assessment. Overall, the rate of participants not responding to the assessment at T1 was 22%, at T2, it was 38%, at T3, it was 42%, and 46% at T4 (Fig. 1).

### Baseline sample characteristics

Demographic and bereavement characteristics of participants are summarized in Table 1.

**Table 1:** Demographic and bereavement characteristics of participants

Characteristics	Total Sample (*n* = 50)	
	*n*	%
Female	33	66
Age in years*	41.2 (12.1) [24–71]	
Marital status		
Single	26	52
Married/partnered	12	24
Divorced/separated	11	22
Widowed	1	2
University education	39	78
Employed	36	72
Active member of a religious organization	3	6
Caucasian	38	77.6
First-degree relationship to deceased	39	78
Unnatural death (suicide, homicide, accident)	19	38
Years since loss		
0 to 3 years	11	22.9
4 to 7 years	19	39.6
8 to 15 years	9	18.8
More than 15 years	9	18.8
Previous therapy for grief	27	54
Previous medication for grief	14	28
Previous ayahuasca for grief	10	20

*Data are expressed as mean (SD) [range]

The quality of the relationship with the deceased was positive for 68%, negative for 12%, and ambiguous for 20%. For 82% of participants, the death was considered a traumatic event and 14% were unsure. Among the sample, 68% had lost an average of 4.11 (*SD* = 2.15) significant people in the past (range 2–10).

The main motivations for attending the retreat were therapeutic (34.1%), spiritual growth (34.1%), and personal development (31.7%). None of the participants affirmed a recreational purpose, such as drug tourism. Among the sample, 76% expected that the ayahuasca ceremonies at the center would improve their grief symptoms and 24% were not sure. Of the overall sample, 70% had never used ayahuasca before. Of the remaining 30% of the sample (*n* = 15), 40% drank ayahuasca less than 3 times, 40% between 3 and 7 times, and 20% more than 7 times.

### Setting variables

Participants attended an average of 18.1 (*SD* = 8.7) days at the center (range 9–30), where they participated in an average of 6.62 (*SD* = 1.37) ayahuasca ceremonies (range 4–9). Of the 39 participants who responded at T1, 53.8% perceived ayahuasca as the most relevant factor in the evolution of their grief symptoms during their stay at the center, followed by the work of the Shipibo healers (35.9%), being with other guests (5.1%), the experience of being in the jungle (2.6%), and having attended other alternative activities, such as yoga classes (2.6%). Moreover, 78.4% described an ayahuasca experience that directly affected their grief process.

### Main outcome analyses

The results for outcome variables at all assessment points (T0, T1, T2, T3, and T4) are presented in Table 2.

**Table 2:** Outcome of repeated measures at baseline (T0) and each time point during follow-up. Values are given as means (with standard deviations)

Measures	T0 Mean (SD) *N* = 50	T1 Mean (SD)/Sig.^a†^ *N* = 39	T2 Mean (SD)/Sig.^b†^ *N* = 31	T3 Mean (SD)/Sig.^c†^ *N* = 29	T4 Mean (SD)/Sig.^d†^ *N* = 27
TRIG	40.5 (9.6)	31.1 (9.3)***	29.7 (9.8)***	31.8 (8.9)***	29.7 (9.1)***
SA-45					
Hostility	59.0 (5.6)	55.2 (4.1)***	55.9 (4.3)*	57.4 (5.5)	56.8 (4.6)
Somatization	62.3 (9.3)	57.5 (6.6)***	56.0 (8.3)***	56.9 (7.9)**	57.9 (9.0)***
Depression	62.0 (6.9)	55.3 (8.5)***	56.0 (6.8)**	57.0 (6.3)*	57.0 (9.0)**
Obsess. compulsive	63.9 (8.5)	56.9 (7.4)***	56.4 (8.0)***	57.1 (7.8)***	58.1 (9.2)**
Anxiety	63.9 (8.5)	54.1 (6.8)***	55.7(7.2)***	56.4 (8.1)**	55.7 (9.7)***
Interp. sensitivity	62.7 (6.7)	55.5 (6.5)***	56.3 (6.4)***	55.8 (7.0)***	55.6 (6.4)***
Phobic anxiety	65.3 (6.4)	63.1 (5.6)	61.6 (4.7)**	61.8 (4.3)*	60.9 (4.9)***
Paranoid ideation	59.0 (7.7)	53.0 (6.7)***	53.2 (6.8)***	54.8 (7.5)*	54.1 (7.9)**
Psychoticism	63.4 (5.3)	60.5 (3.6)**	59.9 (3.4)***	60.0 (3.2)**	60.2 (3.5)***
GSI	63.2 (7.7)	54.2 (7.9)***	53.7 (8.9)***	54.8 (8.8)***	55.1 (9.0)***
WHOQoL					
Physical health	14.7 (2.9)	16.5 (2.8)***	17.0 (2.2)***	16.6 (2.2)***	16.2 (2.6)**
Psychological health	12.6 (2.9)	15.6 (2.4)***	15.5 (2.2)***	15.6 (1.5)***	14.7 (2.5)**
Social relationships	12.3 (3.2)	15.0 (3.9)***	14.8 (3.1)**	15.4 (3.1)**	14.1 (3.6)**
AAQ-II	27.0 (7.0)	18.8 (5.7)***	18.7 (5.7)***	19.4 (5.6)***	19.4 (6.7)***
EQ-Delefting	35.3 (7.3)	42.5 (6.2)***	43.3 (5.7)***	42.6 (4.9)***	43.3 (6.6)***

^†^Bonferroni correction is performed

^**a**^*p* value calculated by paired sample *t* test from T0 to T1 (*N* = 39)

^**b**^*p* value calculated by paired sample *t* test from T0 to T2 (*N* = 31)

^**c**^*p* value calculated by paired sample *t* test from T0 to T3 (*N* = 29)

^**d**^*p* value calculated by paired sample *t* test from T0 to T4 (*N* = 27)

Asterisks indicate *p* values: *p < .05; **p < .01; ***p < .001 in the paired sample *t* tests

**Fig. 2:**
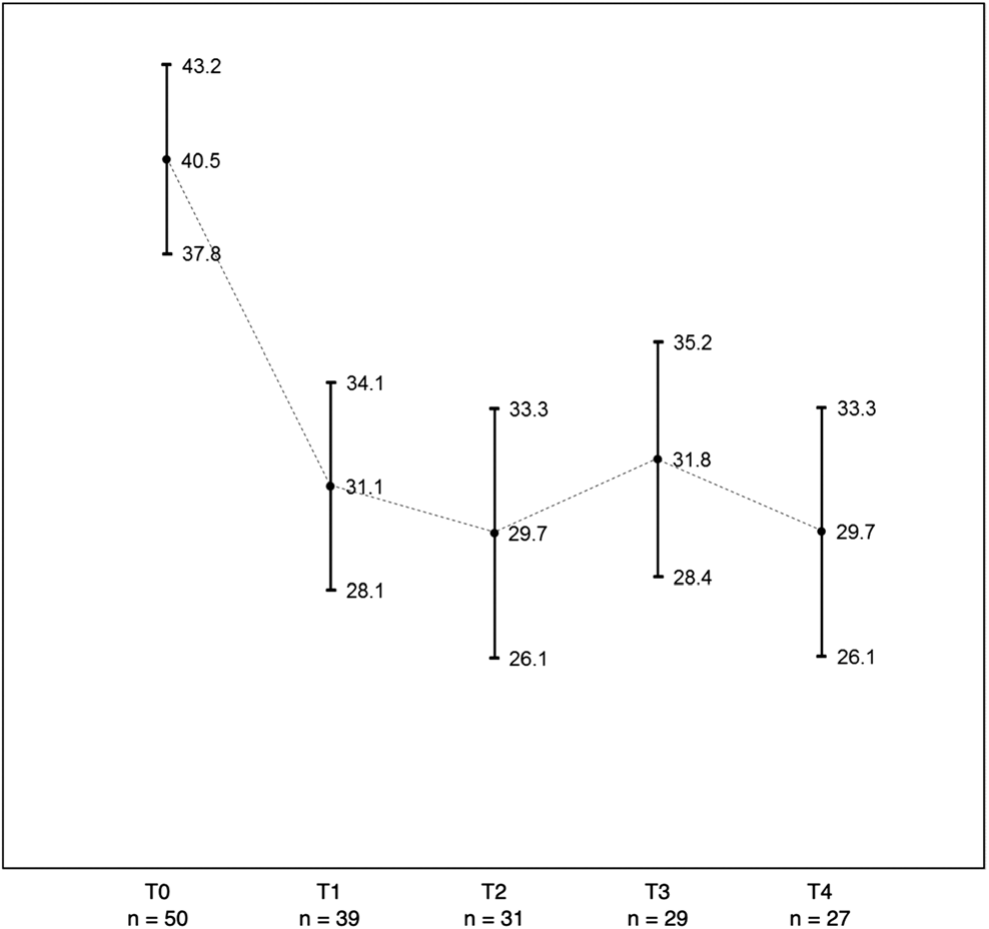
Graph of the mean values (dots) and confident intervals (CI, 95%) of the severity of grief at baseline (T0) and follow-up (T1, T2, T3, and T4)

TRIG score was significantly reduced from baseline to all point times follow-up assessment (T1, T2, T3, and T4: *p* < 0.001). This significant improvement in the severity of grief is shown graphically with confidence intervals (95% CI) (Fig. 2). Calculating effect sizes according to Cohen’s method, we found a high effect size at all assessments points (T1 = 0.84, 95% CI = 0.45–1.23; T2 = 1.38, 95% CI = 0.92–1.80; T3 = 1.16, 95% CI = 0.72–1.64; and T4 = 1.39, 95% CI = 0.93–1.88).

Similarly, SA-45 showed a significant improvement on the Global Severity Index (GSI) for all assessments (T1, T2, T3, and T4: *p* < 0.001), showing a high effect size at T1 = 1.11, 95% CI = 0.77–1.45; T2 = 1.06, 95% CI = 0.73–1.39; T3 = 0.90, 95% CI = 0.59–1.22; and T4 = 1.1, 95% CI = 0.68–1.52. Almost all of the psychopathological subscales of SA-45 showed a significant decrease after the retreat (T1: *p* ≤ 0.003), which were maintained over the follow-up assessments (T2: *p* ≤ 0.018; T3: *p* ≤ 0.017; and T4: *p* ≤ 0.005) with the exception of hostility at T3 (*p* = 0.451) and T4 (*p* = 0.087). The only subscale that did not show a significant decrease after the retreat was phobic anxiety (T1: *p* = 0.062), although a significant reduction in this symptom was observed over subsequent assessments (T2: *p* = 0.002; T3: *p* = 0.016; and T4: *p* < 0.001).

Finally, the Symptom Assessment-45 Questionnaire (WHOQoL-BREF) questionnaire also showed significant improvements in physical health, psychological health, and social relationship scales after the retreat (T1: *p* < 0.001). The improvements in physical health were maintained at T2, T3, and T4 (*p* < 0.001); in psychological health at T2, T3, and T4 (*p* < 0.001); and in social relationships at T2 (*p* = 0.003), T3 (*p* = .001), and T4 (*p* = 0.004). The effect size was larger for the psychological health subscale (T1 = 0.92, 95% CI = 0.056–1.26; T2 = 0.89, 95% CI = 0.54–1.27; T3 = 0.82, 95% CI = 0.53–1.11; and T4 = 0.73, 95% CI = 0.33–1.08).

Both mechanisms of change, experiential avoidance (AAQ-II) and decentering (EQ-Decentering), significantly improved after the retreat (T1: *p* < 0.001), maintaining these gains at all follow-up assessments (T2, T3, and T4: *p* < 0.001). AAQ-II showed a high effect size for all assessments, as T1 = 0.90, 95% CI = 0.62–1.18; T2 = 1.09, 95% CI = 0.72–1.48; T3 = 1.14, 95% CI = 0.80–1.51; and T4 = 1.07, 95% CI = 0.57–1.53. EQ-Decentering also showed a high effect size for all assessments, as T1 = 0.96, 95% CI = 0.64–1.28; T2 = 0.99, 95% CI = 0.55–1.43; T3 = 0.93, 95% CI = 0.46–1.36; and T4 = 1.26, 95% CI = 0.86–1.66.

### Bereavement adjustment over time

The ayahuasca experiences at the center had a positive effect on grief symptoms for 92.3% of the sample, for 5.1%, it had no influence and 2.6% experienced a negative effect on their grief process. The bereavement adjustment of the participants at all follow-up assessments, the uses of therapeutic resources to treat grief, and the persistent benefits and adverse effects derived from the ayahuasca experiences at the center are shown in Table 3.

**Table 3:** Bereavement adjustment, adverse effects, and benefits of ayahuasca at each time point during follow-up

	T1 *N* = 39 *n* %		T2 *N* = 31 *n* %		T3 *N* = 29 *n* %		T4 *N* = 27 *n* %	
Perception in relation to grief								
I feel worse	1	2.6	0	0.0	0	0.0	1	3.7
I feel the same	2	5.1	2	6.5	1	3.4	0	0.0
I feel better	36	92.3	29	93.5	28	96.6	26	96.3
Persistent adverse effects of ayahuasca^a^								
Physical health	0	0.0	0	0.0	0	0.0	0	0.0
Mental health	0	0.0	0	0.0	0	0.0	1	3.7
Social relationships	1	2.6	1	3.2	0	0.0	1	3.7
Spirituality	0	0.0	0	0.0	0	0.0	1	3.7
Persistent benefits of ayahuasca^a^								
Physical health	30	76.9	19	61.3	18	62.1	17	63.0
Mental health	34	87.2	26	83.9	24	82.1	24	88.9
Social relationships	26	66.7	17	54.8	19	65.5	18	66.7
Spirituality	33	84.6	26	83.9	26	89.7	24	88.9
Therapy to treat grief			4	12.9	8	27.6	9	33.3
Medication to treat grief			0	0.0	0	0.0	1	3.7
Ayahuasca to treat grief			3	9.7	4	13.8	4	14.8

^a^Multiple choice responses

### Correlation analysis between grief and mechanism of change

**Fig. 3:**
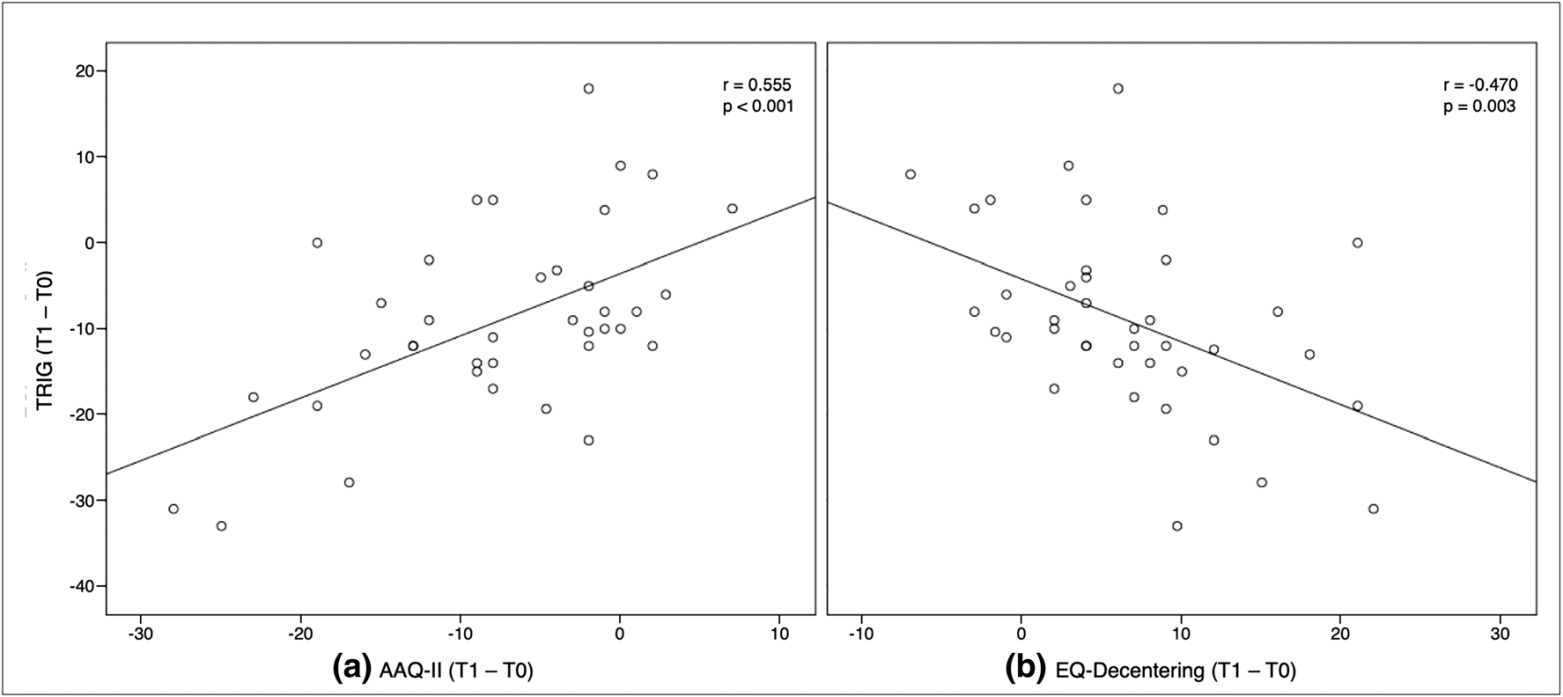
Scatter plot of the relationship between the severity of grief (TRIG) and experiential avoidance (AAQ-II) (**a**) and decentering (EQ-Decentering) (**b**)

The significant decrease in experiential avoidance (AAQ-II) between T0 and T1 was found to be associated with the significant decrease in the severity of grief (TRIG) between T0 and T1 (*r* = 0.55; *p* < 0.01). In addition, the correlation between the significant improvement in decentering ability (EQ-Decentering) between T0 and T1 and the decrease in grief symptomatology (TRIG) between T0 and T1 (*r* = −0.47) was also significant (*p* < 0.01) (Fig. 3).

### Subgroup analysis

Of the 39 participants who completed the follow-up assessment at T1, a subgroup of 9 participants who completed an additional assessment 2 months prior to the retreat (T-1) were elegible for the subgroup analysis (ANOVA). The homogeneity of the groups was tested by comparing key variables (baseline characteristics, primary and secondary outcome measures). No significant differences were found in baseline sample characteristics nor outcome variables (*p* > 0.05). The average number of days registered in the subsample between T-1 and T0 was 45.0 (*SD* = 3.87), and between T0 and T1, it was 47.67 (*SD* = 8.38). ANOVA revealed a significant main effect of time on the TRIG [*F* (2; 16) = 11.18, *p* = 0.001]. Post hoc tests showed a significant improvement only between T1 and T0 [− 13.26 (*SD* = 11.17); *p* = 0.022] and not between T0 and T-1 [− 1.07 (*SD* = 5.24); *p* > 0.999], indicating that the reduction in the severity of grief was only observed during participants’ stays at the center.

Similarly, ANOVA revealed a significant main effect of time on secondary variables during participants’ stays at the center. This is the case with the GSI of the SA-45 questionnaire [*F* (1,4: 10,9) = 10.30; *p* = 0.006], only between T1 and T0 [− 8.78 (*SD* = 7.74); *p* = 0.028] and not between T0 and T-1 [0.33 (*SD* = 3.08); *p* > 0.999], and with psychological health of the WHOQol-BREF (*F* (1,2; 9,8) =12.28; *p* = 0.004), only between T1 and T0 [2.52 (1.97); *p* = 0.015] and not between T0 and T-1 [0.03 (2.33); *p* > 0.999].

Finally, AAQ-II also showed a significant main effect of time [*F* (2,14) = 7.45; *p* = 0.006], only between T1 and T0 [− 9.88 (*SD* = 8.59); *p* = 0.042] and not between T0 and T-1 [3.25 (*SD* = 4.74); *p* = 0.281], as well as EQ-Decentering [*F* (1,4:10,8) = 7.30; *p* = 0.015], only between T1 and T0 [7.30 (*SD* = 6.29); *p* = 0.025] and not between T0 and T-1 [− 1.47 (*SD* = 3.25); *p* = 0.638].

## Discussion

This is the first study to prospectively assess the long-term effects of ayahuasca in participants grieving the death of a loved one. In this observational study, the primary result showed that the severity of grief significantly decreased after drinking ayahuasca, being maintained over 1 year of follow-up. The subgroup analysis performed in a representative subsample allowed us to infer that the significant differences in outcomes are due to the effect of their stay at the center and not the passing of time. The large effect size obtained at all point assessments on the primary scale clearly exceeds the mean effect size post-intervention (Hedges’s *g* = 0.41) and at follow-up (*g* = 0.45) obtained in the last meta-analysis focused on the effectiveness of current treatments developed to treat complicated grief and prolonged grief disorder (PGD) (Johannsen et al. 2019). Studies that have obtained higher effects sizes have applied specific protocols of intervention, which involved conducting from 12 to 25 psychotherapeutic sessions during periods of 3 to 9 months, or even longer (Bryant et al., 2014; Rosner et al. 2014; Boelen et al. 2007). We dutifully note that the Temple of the Way of Light is not conducting a specific program to treat grief symptoms, and that the maximum duration of the retreat was 1 month, during which ayahuasca was taken a maximum of 9 times.

It is well known that grief symptoms usually present a high rate of comorbidity with mood and anxiety disorders, influencing the quality of life of bereaved adults (Boelen and Prigerson 2007). In our sample, almost all the scales in SA-45 were above the score of 60 at baseline (percentile of 84) (Davison et al. 1997) suggesting likely problems with somatization, depression, obsessive-compulsiveness, anxiety, interpersonal sensitivity, phobic anxiety, and psychoticism. With the exception of phobic anxiety, all of these symptoms decreased significantly at all time points over follow-up to a level that is considered within the normal range in a non-patient normative sample (Davison et al. 1997). These results are in line with previous studies that found lower levels of psychopathology after drinking ayahuasca in first-time ayahuasca ceremony participants (Barbosa et al. 2009), as well as in long-term users as compared with control groups (Bouso et al. 2012; Dos Santos et al. 2016). Given that phobic anxiety is expressed through symptoms of uneasiness when in open spaces and crowds, the fact that we did not find significant differences in this scale after the retreat, but found differences throughout later follow-up assessments (T2, T3, and T4), could indicate a need for a period of time to adapt to the city environment after being in the jungle. However, phobic anxiety and psychoticism are on the threshold of being considered problematic areas, according to the follow-up assessments. As far as we know, this is the first article to look deeply into these psychopathological symptoms among a sample of grievers, so we cannot compare many of these subscales with other studies. Only one previous study has assessed a few of these psychopathological symptoms after participants received prolonged grief-specific cognitive-behavioral therapy (Rosner et al. 2014), an integrative cognitive-behavioral approach that includes structured exposures and cognitive restructuring. In this study, there were no differences in terms of phobic anxiety, somatization, and anxiety when participants were compared with a waiting list group.

The significant long-term improvements on all the scales of quality of life in our sample offer a broader picture of the impact of ayahuasca on the evolution of grief. Previous studies have found similar results in first-time ayahuasca ceremony participants (Barbosa et al. 2009), as well as improvement in psychosocial well-being in regular participants (Bouso et al. 2012; Dos Santos et al. 2016). However, last meta-analysis on psychological interventions for grief did not find statistically significant effects on health-related quality of life (Johannsen et al. 2019).

Although subjective responses about the persistent harms and benefits of ayahuasca experiences at the retreat support the overall outcomes, we must bear in mind that a large proportion of the participants expected that ayahuasca ceremonies would improve their grief symptoms, which could promote suggestibility and enhance positive outcomes. The fact that most of them affirmed that ayahuasca had a positive effect in terms of mental health and spirituality led us to ponder the role that spiritual coping played in the adjustment of bereavement in our sample. Previous studies have shown that spirituality can play a centrally important role in helping individuals adapt to the loss of a loved one (Wortmann and Park 2008; Hays and Hendrix 2008).

In our sample, 78.4% of participants described an ayahuasca experience that directly affected their grief process. Although the content analysis of participants’ ayahuasca experiences is pending publication, a previous study described emotional confrontations with the reality of the death, the reviewing of biographical memories, and a reencounter with the deceased, as common ayahuasca experiences among grievers (González et al. 2017). The pharmacological effects of ayahuasca have been described extensively (Dos Santos et al. 2012, 2013; Riba et al. 2001, 2011), including a dose-dependent curve with a slow onset during the first 45 min, a peak in the experience at around 90 min, and slow decrease in the effects from 150 to 250 min. Under the effects of a medium or high dose, the peak of the experience could be similar to the immersion used in virtual reality exposure therapy (VRET), which allows individuals to bypass symptoms of experiential avoidance (Gonçalves et al. 2012). Other studies have compared acute ayahuasca effects with a controlled exposure to autobiographical material (Domínguez-Clavé et al. 2016). This acute confrontation with difficult memories, thoughts, and feelings could promote increased acceptance, leading to psychological modifications that are observable beyond the time frame of the acute inebriation (Soler et al. 2018). Although this is the only study that has assessed the modulation of acceptance (or experiential avoidance) after an ayahuasca experience over the course of 1 year, our results are in line with other studies that have found an increase in acceptance to result from an ayahuasca experience (Domínguez-Clavé et al. 2016; Soler et al. 2018). Nevertheless, around 150 min after ayahuasca intake, the intensity of the effects begins to decrease, allowing a healthy distance from the psychological material and leading to a reflective phase (Kjellgren et al. 2009). During this phase, decentering could improve (Fresco et al. 2007). Gains in the ability to decenter are in line with other studies that have found an increase in decentering abilities following ayahuasca intake (Domínguez-Clavé et al. 2016; Soler et al. 2016; Sampedro et al. 2017), and in those with more than 15 ayahuasca experiences compared with those with non or few experiences (Franquesa et al. 2018). However, contrary to a previous study (Sampedro et al. 2017), the gains in decentering in our sample persisted over a year of follow-up.

These results are also in line with previous studies that show a positive relationship between high levels of acceptance and high levels of decentering (Hayes et al. 2017). In our sample, we observe that increased acceptance and decentering following the retreat have a significant correlation with improvements in the severity of grief. Acceptance is considered a key mechanism of change in complicated grief treatment (Glickman et al. 2017), a form of cognitive-behavior therapy with elements of interpersonal psychotherapy and motivational interviewing (Shear et al. 2016). On the other hand, decentering is a mediator in the metacognitive approach to treating grief (Wenn et al. 2015), a form of psychotherapy that uses detached mindfulness to alter dysfunctional thinking styles by helping individuals to understand their “thoughts about thoughts.” Accommodation (new learning) is a psychological process that has been associated with an ability to engage with traumatic experiences when it is combined with more acceptance and decentering (Hayes et al. 2017). Accommodation provides an opportunity for constructive processing and meaning making regarding the experience (Neimeyer 2019). Moreover, other kinds of experiences, such as having a reencounter with the deceased (González et al. 2017), could modulate the internal working model of the deceased and the attachment style maintained with him or her after death. Future studies should explore more potential mechanisms of change in order to reach a better understanding of the therapeutic potential of ayahuasca among the bereaved.

The study has some limitations. First, the naturalistic design of the study does not allow us to isolate the effects of ayahuasca from the other diverse variables in the setting, which include the healing work of the Shipibo shamans during the ayahuasca ceremony. In fact, the separation of the effects of ayahuasca from the healer’s work is not conceived of within the Shipibo traditional medicinal framework, in which participants drank the ayahuasca. Moreover, without a placebo group it is not possible to know if ayahuasca was truly responsible for the improvement in grief symptoms, as it is not possible to draw conclusions about causality. Second, the lack of scientific evidence on the stability of ayahuasca alkaloids over time does not guarantee similar concentrations when participants took ayahuasca in the center and at the time, the samples were analyzed. Moreover, the lack of knowledge about the doses used throughout the ceremonies by each participant prevents us from assessing to relate the improvement in the severity of grief in relation with the doses of DMT or betacarbolinnes ingested. Third, the sample size is relatively small and there were a large number of participants that did not complete all the follow-up assessments. This limitation can be extended to the subgroup analysis, which was conducted to control for the effect of time passing. Fourth, nine participants attended psychotherapy over the year follow-up, four took ayahuasca again to treat grief symptoms, and one participant took medication, so we cannot attribute the maintenance of the improvement in grief symptoms exclusively to the persistent benefits of the ayahuasca experiences at the retreat. Fifth, we did not assess Prolonged Grief Disorder or Persistent Complex Bereavement Disorder, having used self-report measures rather than clinical interviews. This, along with the fact that the sample was heterogeneous, in terms of the causes of loss and the amount of time that had passed since the loss, makes the extent to which subgroups profited differently from ayahuasca uncertain. Lastly, we used a direct method to recruit participants who were willing to participate in ayahuasca sessions, which may limit the generalizability of our results to treatment-seeking bereaved people in general or whose able to respond an online survey. To reach stronger conclusions, large controlled cohort studies or randomized, placebo-controlled trials conducted with adequate samples of participants will provide evidence of causality.

## Conclusions

This study offers preliminary findings in support of the long-term therapeutic potential of ayahuasca for bereavement. A significant improvement in the severity of grief and the high effect size maintained over a year follow-up are sufficiently encouraging to warrant further research. Furthermore, the assessment of experiential avoidance and decentering highlights the improvement of these mechanisms of change following drinking ayahuasca and their relation to grief improvement. Therefore, more research is necessary to better understand ayahuasca’s therapeutic value in terms of relief from grief symptoms.

## Electronic supplementary material

ESM 1 (DOCX 108 kb)
