# Supplementary material for: Therapeutic potential of ayahuasca in grief: a prospective, observational study
Source: Psychopharmacology (Berl). 2020 Jan 14;237(4):1171–82. doi: 10.1007/s00213-019-05446-2 (PMC7113212; doi:10.1007/s00213-019-05446-2)
Supplement: Supplementary file 1 — (DOCX 108 kb) [file 213_2019_5446_MOESM1_ESM.docx]

**Supplementary Online Content**

This supplementary material has been provided by the authors to give readers additional information about their work.

**eAppendix: Questions included in Bereavement Adjustment Questionnaire over T1, T2, T3 and T4 follow-up assessments.**

Thank you for deciding to collaborate with us, helping us to advance the scientific knowledge about the potential risks and benefits of ayahuasca.

With each question you answer you are providing unique and singular knowledge that only you can offer, as it is from your own personal reflections, feelings, thoughts and emotions. For this reason, we ask that you answer each question with the utmost sincerity.

Our hope is that the greatest number of people possible will be able to benefit from this study in the future.   Thank you very much for your help and collaboration!

**Bereavement Adjustment Questionnaire at T1**

1. How do you feel with regards to your grief since you have returned from the Temple of the Way of Light?

- I feel worse
- I feel the same
- I feel better

1. How do you think the ayahuasca you drank at the Temple of the Way of Light has affected your grief?

- It has had a positive effect on my grief
- It had no influence on my grief
- It has had a negative effect on my grief

1. Has your use of ayahuasca at the Temple of the Way of Light brought with it any harm, injury or adverse effect?

- Yes (If the answer was affirmative 3a)
- No

3a. Which area(s) of your life have been affected?

- Physical health
- Mental health
- Social relationship
- Spirituality

1. Has your use of ayahuasca at the Temple of the Way of Light brought with it any benefit?

- Yes (If the answer was affirmative 4a)
- No

4a. Which area(s) of your life have been affected?

- Physical health
- Mental health
- Social relationship
- Spirituality

**Bereavement Adjustment Questionnaire at T2, T3 and T4**

1. How do you feel with regards to your grief since you have returned from the Temple of the Way of Light?

- I feel worse
- I feel the same
- I feel better

1. Has your use of ayahuasca at the Temple of the Way of Light brought with it any harm, injury or adverse effect which persist at present?

- Yes (If the answer was affirmative 2.a)
- No

2.a. Which area(s) of your life have been affected?

- Physical health
- Mental health
- Social relationship
- Spirituality

1. Has your use of ayahuasca at the Temple of the Way of Light brought with it any benefit which persist at present?

- Yes (If the answer was affirmative 3.a)
- No

3.a. Which area(s) of your life have been affected?

- Physical health
- Mental health
- Social relationship
- Spirituality

4. Have you received psychological therapy for your grief since you arrived from the Temple of the Way of Light?

- No
- Yes: ^1*^What kind of therapy have you done?: ___

**5.** Have you taken any medication to treat your grief since you arrived from the Temple of the Way of Light?

- No
- Yes:^2*^What kind of medication?:__

**6**. Have you taken more ayahuasca to treat your grief since you arrived from the Temple of the Way of Light?

- No
- Yes

**^*1^** Affirmative responses include:

*cognitive-behavioral therapy, acceptance and commitment therapy, psychodinamic psychotherapy and psychotherapy (in general).*

It has been excludeded:

*support groups, coaching, energetic therapies and accupunture.*

**^*2^** Affirmative responses include:

*antidepressant medication.*

It has been excluded:

*food supplements, herbs and other psychedelics.*
